# Comparison of methods for the isolation of human breast epithelial and myoepithelial cells

**DOI:** 10.3389/fcell.2015.00032

**Published:** 2015-05-21

**Authors:** Arantzazu Zubeldia-Plazaola, Elisabet Ametller, Mario Mancino, Miquel Prats de Puig, Anna López-Plana, Flavia Guzman, Laia Vinyals, Eva M. Pastor-Arroyo, Vanessa Almendro, Gemma Fuster, Pedro Gascón

**Affiliations:** ^1^Department of Medical Oncology, Hospital ClínicBarcelona, Spain; ^2^Department of Medicine, University of BarcelonaBarcelona, Spain; ^3^Institut d'Investigacions Biomediques August Pi i SunyerBarcelona, Spain; ^4^Department of Senology, Clínica PlanasBarcelona, Spain; ^5^Histopathology-Citology, Anatomical Pathology Service, Centro Medico TeknonBarcelona, Spain; ^6^Kidney and Acid-base Physiology Research Group, Institute of Physiology, University of ZurichZurich, Switzerland; ^7^Division of Medical Oncology, Department of Medicine, Harvard Medical School, Dana-Farber Cancer Institute, Brigham and Women's HospitalBoston, MA, USA

**Keywords:** epithelial cells, myoepithelial cells, isolation, primary cell culture

## Abstract

Two lineages, epithelial, and myoepithelial cells are the main cell populations in the normal mammary gland and in breast cancer. Traditionally, cancer research has been performed using commercial cell lines, but primary cell cultures obtained from fresh breast tissue are a powerful tool to study more reliably new aspects of mammary gland biology, including normal and pathological conditions. Nevertheless, the methods described to date have some technical problems in terms of cell viability and yield, which hamper work with primary mammary cells. Therefore, there is a need to optimize technology for the proper isolation of epithelial and myoepithelial cells. For this reason, we compared four methods in an effort to improve the isolation and primary cell culture of different cell populations of human mammary epithelium. The samples were obtained from healthy tissue of patients who had undergone mammoplasty or mastectomy surgery. We based our approaches on previously described methods, and incorporated additional steps to ameliorate technical efficiency and increase cell survival. We determined cell growth and viability by phase-contrast images, growth curve analysis and cell yield, and identified cell-lineage specific markers by flow cytometry and immunofluorescence in 3D cell cultures. These techniques allowed us to better evaluate the functional capabilities of these two main mammary lineages, using CD227/K19 (epithelial cells) and CD10/K14 (myoepithelial cells) antigens. Our results show that slow digestion at low enzymatic concentration combined with the differential centrifugation technique is the method that best fits the main goal of the present study: protocol efficiency and cell survival yield. In summary, we propose some guidelines to establish primary mammary epithelial cell lines more efficiently and to provide us with a strong research instrument to better understand the role of different epithelial cell types in the origin of breast cancer.

## Introduction

The mammary gland is a tubuloalveolar structure consisting of a branching network of ducts ending in terminal ductal lobular units (TDLUs) that constitute the functional domains of the pre-menopausal breast. These ducts are composed of two continuous layers of primary epithelial lineages: the inner part is comprised of luminal epithelial cells that can produce milk during lactation; the outer layer contains myoepithelial cells that provide the ducts with contractile ability (Almendro and Fuster, 2011; Fu et al., 2014). These two main cell populations are surrounded by a basal membrane and are embedded in an extracellular matrix composed of different cell types such as macrophages, fibroblasts, adipocytes, endothelial cells, and other cells from the immune system, which together constitute the microenvironment (Almendro and Fuster, 2011; Fu et al., 2014).

The epithelial mammary gland compartment drives the mammary gland dynamics during a women's lifetime, and is also responsible for most breast cancers. Although breast cancers mainly originate in the epithelial lineage, the myoepithelium also plays a key role in tumor progression by controlling the invasiveness potential of the tumor cells (Hu et al., 2008; Fu et al., 2014). Therefore, there have been significant efforts to optimize the protocols used to isolate and characterize the main breast cell populations (Speirs et al., 1998; Gudjonsson et al., 2004; Stingl et al., 2005; Shipitsin et al., 2007; Labarge et al., 2013; Raouf and Sun, 2013). The most extensively used techniques include the isolation of cell subpopulations by phenotypic markers or by functional means. Epithelial and myoepithelial mature cells can be isolated on the basis of differential expression of some cell surface markers, such as CD227, EpCAM, CD44, and CD24 for epithelial cells, and CD10 for the myoepithelial lineage (Fu et al., 2014). Additional markers can be used to isolate estrogen receptor negative mammary stem cells and lineage-restricted progenitors (Tosoni et al., 2012). However, culture conditions could influence the expression pattern of some of these cell-specific markers, and therefore it is necessary to characterize the markers that are robust enough for proper identification and isolation of cell subpopulations (Gudjonsson et al., 2002; Tosoni et al., 2012). Another approach to the isolation of mammary stem cells is based on two of their functional properties in comparison with their progeny, instead of markers (Tosoni et al., 2012). In fact, Tosoni et al. used mammary stem cells' quiescent or slowly proliferative behavior and growth capacity in anchorage-independent conditions to identify them (Tosoni et al., 2012).

An important challenge in the isolation of breast epithelial and myoepithelial cells is posed by the tissue digestion and fractionation procedure. Several papers have been published describing methods to obtain mammary epithelial cell lines from fresh tissue (Speirs et al., 1998; Gudjonsson et al., 2004; Stingl et al., 2005; Shipitsin et al., 2007; Labarge et al., 2013; Raouf and Sun, 2013). Approaches vary in the mechanical manipulation (discarding adipose tissue or not, the size of pieces), digestion (time of digestion, type/concentration of enzymes added such as collagenase, hyaluronidase or a combination of both), cell fraction separation (by sequential filtering or differential centrifugation), and final cell isolation (by immunomagnetic beads or by sorting) used to isolate the mammary epithelial cells (Speirs et al., 1998; Gudjonsson et al., 2004; Stingl et al., 2005; Shipitsin et al., 2007; Labarge et al., 2013; Raouf and Sun, 2013). Each of these procedures differs in the cell yield and viability due to the digestion, the fractionation steps, and the cell culture. Moreover, primary cultures have some other limitations such as the appearance of senescence after 10–40 population doublings, which hinders their long-term culture. The type of medium and/or the addition of Rho-associated protein kinase (ROCK) inhibitor after digestion can delay the senescence process, improve cell proliferation (Hammond et al., 1984; Garbe et al., 2009) and prevent anoikis phenomena. Therefore, the technical problems described to date affect cell viability and yield, hamper work with primary cells, and indicate the need to optimize technology for the proper isolation of epithelial and myoepithelial cells. Here, we tested in parallel four protocols to establish epithelial and myoepithelial cells in culture, starting from surgically resected breast tissue. Based on our ability to more effectively obtain viable mammary cells, we recommend overnight digestion with a lower concentration of enzymes (slow digestion) followed by differential centrifugation. In addition, we strongly suggest that ROCK inhibitor should be added to the media to avoid anoikis and early senescence.

## Materials and reagents

- Dulbecco's Modified Eagle Medium: Nutrient Mixture F-12 (DMEM/F12) + PSF (Penicillin, Streptomycin and Fungizone) (Gibco, Life Technologies) (for the digestion mixture)
- Sterilized surgical material (scalpels, scissors, forceps)
- Sterile petri dishes or metal surface to chop the tissue
- Blender (Bowl volume: 2 L; Bowl size: 17 cm high × 14 cm diameter)
- 1 L sterile glass bottle
- 2 L beaker
- BSA (Bovine Serum Albumine) (Sigma)
- Collagenase type IV (Sigma C-5138)
- Hyaluronidase (Sigma H-3506)
- Cell culture dishes and tubes
- PBS (Phosphate Buffered Saline) (Gibco, Life Technologies)
- 500 μm Filters (Corning)
- 250, 100, 40, and 20 strainers (Millipore)
- Fibroblasts culture medium: DMEM + 10% Fetal Bovine Serum (FBS) + 1% PSF + Glutamax (Gibco, Life Technologies)
- Epithelial and organoid culture medium: M87A [formula previously described (Garbe et al., 2009)]
- ROCK inhibitor or Y27632 (Sigma Y-0503)
- 0.25% Trypsin-EDTA (Life Technologies)

## Methods

### Human subjects

The human protocol was approved by each institutional review board. Fresh healthy mammary tissue from women between 20 and 60 years old was obtained from 13 patients who underwent reduction mammoplasty and 2 patients who had a mastectomy after breast cancer in the contralateral breast. The samples were obtained under the approval of the Institutional Review Board of the Hospital Clinic and Clinica Planas in Barcelona.

### Breast epithelial and myoepithelial cell isolation

#### Tissue digestion

Between 80 and 150 g of fresh mammary tissue was used as starting material for the tissue digestion. Samples had to be handled in sterile conditions. In some cases, samples were kept at 4°C for 24 h before being processed, a step that slightly reduced the efficiency of the methodology. Fatty tissue was manually separated from breast areas that were rich in ducts, using sterile scalpels and forceps (Stingl et al., 2005). Subsequently, the epithelial-enriched tissue was minced into small pieces (the size depended on the procedure used and is described for each method below) with the help of sterile scalpels, scissors, and forceps.

**Figure 1:**
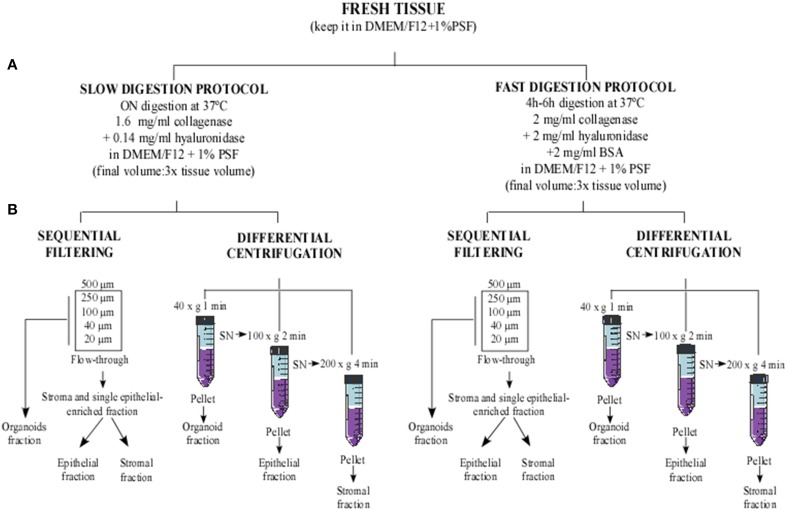
**Flowchart of methodological approaches used to obtain cellular fractions from human reduction mammoplasty tissue**. Human tissue was minced into small fragments and sequentially digested using two digestion protocols. **(A)** Slow digestion (overnight at low enzymatic concentration) and fast digestion (4–6 h at high enzymatic concentration). Afterwards, the digested tissues obtained from each method were processed using two cell separation techniques: **(B)** sequential filtering and differential centrifugation.

For our purpose, the tissue digestion was performed following the two methods described below (Figure 1), and each sample was distributed as shown in Table S1. When two methods were performed, the samples were equally divided (40–75 g in each method).

#### Fast digestion

The breast tissue free of adipose areas was minced into 6–7 mm pieces and placed in a new sterile dish with Dulbecco's Modified Eagle's Medium—Ham's F12 (DMEMF12), supplemented with 100 U/mL penicillin, 100 μg/mL streptomycin and 5 μg/mL Fungizone (PSF) to prevent the tissue from drying out. Additionally, breast tissue pieces were chopped and grinded in the presence of supplemented DMEMF12 medium using a food processor, until the biggest tissue chunks had disappeared and the remaining pieces' size was between 1 and 4 mm.

The processed tissue was placed in a 1 L sterile glass bottle with a stir bar, in the presence of digestion buffer containing supplemented DMEMF12 medium (1% PSF), 250 U/ml of collagenase Type IV, 1400 U/ml of hyaluronidase and 2 mg/mL Bovine Serum Albumin (BSA) (Ince et al., 2007; Shipitsin et al., 2007) until 3 times the tissue volume had been reached. The tissue was then digested at 37°C for 4–6 h in a heated magnetic stirrer at 300 rpm, until only small tissue pieces could still be observed. Next, the digestion status was checked under the microscope. If there were many single cells or many clumps of cells free from attached stroma (organoids), the digestion was stopped by cooling at 4°C.

#### Slow digestion

The epithelial-enriched area was minced into 3–4 mm pieces and placed in a 1 L sterile glass bottle, together with supplemented DMEMF12 medium with 200 U/ml of collagenase Type IV and 100 U/ml of hyaluronidase, until 3X times the tissue volume had been reached. The glass bottle was placed in a shaker approximately at 50 rpm and 37°C overnight (Speirs et al., 1998).

When the digested tissue was homogeneous, the sample was checked under the microscope and if organoids were observed, the digestion was stopped and the cell fraction separation procedure was started. If not, the digestion was continued for 1–2 h more until free organoids were observable under the microscope. In this step, the protocol could be paused by placing the digestion mixture at 4°C for a short period of time before starting the cell fraction separation. If big tissue chunks were still present, the digestion media was collected, filtered through a 500 μm strainer, and kept at 4°C until the fractionation step. New digestion media was added to the remaining undigested tissue and the procedure was monitored until full digestion was achieved. Then, the new digested tissue was mixed with the previous one, and processed in the cell fraction separation step.

#### Cell fraction separation

In order to separate organoids from single epithelial and stromal cells, cell fraction separation was performed using two techniques. The cell fraction separation method described in Table S1 was performed for each sample. Samples were equally divided when they were used for both techniques.

#### Cell fraction separation by sequential filtering

In order to eliminate unprocessed big tissue fragments, digested tissue was poured through 500 μm mesh into 50 mL sterile tubes and centrifuged at 750 g for 10 min at RT. The supernatant was discarded, and the pellets were washed in cold PBS and combined into one 50 mL tube. The washed pellets were centrifuged again at 750 g for 10 min at RT. Pellets were then suspended in PBS and filtered sequentially using 500, 250, 100, 40, and 20 μm cell strainers (Figure 1). In each filter step, the mesh was flushed a couple of times with PBS to prevent the loss of organoids.

The organoid fraction was collected from the upper part of 250, 100, 40, and 20 μm strainers using cold PBS and a 1000 μl pipette (Figure S2). It was important to repeat the collection step from the upper part of the filters several times until the filter was clean, since the organoids could be attached to it, and it could be difficult to collect them (Shipitsin et al., 2007; Labarge et al., 2013).

The stromal and single epithelial enriched fractions were recovered from the flow-through after filtering through the 20 μm strainer (Figure S2). Subsequently, the organoid and stromal single epithelial fractions were centrifuged (Figure S3B). Each pellet was suspended in the desired medium for the subsequent cell culture. M87A medium was used to culture the organoid fraction. Part of the flow-through fraction was suspended in M87A medium to obtain single epithelial cells. The remaining flow-through fraction was suspended in supplemented DMEM medium to promote the growth of stromal cells, such as fibroblasts.

#### Cell fraction separation by differential centrifugation

In this technical approach, three fractions were obtained, corresponding to the organoid fraction, the single epithelial cell enriched fraction, and the stromal cell enriched fraction.

The digested tissue was placed in 50 mL sterile tubes and centrifuged at 40 g for 1 min. The pellet obtained was the organoid fraction (Figure S3B). The supernatant was transferred into new 50 mL tubes and centrifuged at 100 g for 2 min to obtain the pellet as the epithelial fraction (Figure S3B). Finally, the supernatant was put into new 50 mL tubes and centrifuged at 200 g for 4 min to obtain the stromal fraction (Figure 1). The supernatant was then discarded and all the pellets obtained were washed in cold PBS to eliminate digestion remains (Speirs et al., 1998). Organoid and epithelial fractions were suspended in M87A medium and the stromal fraction was suspended in supplemented DMEM medium.

### Cell culture

The organoid and epithelial fractions obtained were suspended in M87A medium (Garbe et al., 2009) and cultured in standard plates. During seeding, approximately 75% of the surface of the plate should be covered by organoids or single cells to obtain optimal efficiency.

In order to avoid anoikis, both organoid and epithelial fractions were cultured in the presence of 10 μm Y27632 dihydrochloride, a ROCK inhibitor competing with ATP for the catalytic domain of the kinase. Y27632 has been described as acting as a promoter of stem cell survival (Watanabe et al., 2007; Koyanagi et al., 2008) and since digestion triggers the loss of both cell-cell and cell-matrix contact derived survival signals, cells are more likely to suffer from apoptosis. Y27632 is a good candidate to avoid this phenomenon.

After 24 h, organoids and single cells started to attach. The seeded cells were maintained without changing the medium for 48–72 h, but additional medium was added. After that, many organoids and single cells still remained in suspension. These cells were seeded again on a new plate. After 24–72 h, organoids and single cells started to attach.

Cells were fed with new medium three times per week and cell confluence was achieved within 5–15 days, depending on the sample and the strategy used.

Cells were frozen in 10% DMSO and 90% M87A medium: approximately 1 million cells in 1 mL of freezing solution.

A critical point is fibroblast contamination in the organoid and epithelial cell fraction. In fact, it is usual to obtain a fibroblast population in these fractions. When fibroblasts are present in the cell culture, differential trypsinization can be used to get rid of them (Halaban and Alfano, 1984). This consists in the faster trypsinization of fibroblasts compared to epithelial and myoepithelial cells. Briefly, cells are incubated with trypsin for a short period of time (less than 1 min), until detachment of fibroblasts is observed. At this point, fibroblasts are discarded and the dish is washed 2–3 times with M87A medium before re-feeding the cells.

### Cell characterization

#### 3D culture and immunofluorescence

The functional characteristics of the epithelial and myoepithelial cells were determined by their ability to form three-dimensional (3D) acinar structures, and the preservation of representative antigens was also evaluated. Briefly, 100,000 cells per well were seeded on top of matrigel in a 24-well plate, as published elsewhere (Debnath et al., 2002). The resulting 3D structures were analyzed by immunofluorescence (Lee et al., 2007; Labarge et al., 2013) of K19 (epithelial cell marker; DSHB, Troma III) and K14 (myoepithelial cell marker; Covance, PRB-155P-100). Images were captured by confocal microscopy.

#### Flow cytometry

Regarding the antigen conservation, cells were examined by flow cytometry, using antibodies against CD227 (epithelial cell lineage; BD Pharmingen, 559774) and CD10 (myoepithelial cell lineage; Biolegend, 312204) as it has been described that their expression is maintained during cell culture using M87A medium (Garbe et al., 2014).

#### qPCR

Fibroblast, lymphocyte and endothelial cell contamination was evaluated by qPCR of Fibroblast Surface Protein (FSP), CD45 and CD146 (Shipitsin et al., 2007) respectively. β-actin was applied as an endogenous control. The primers used were previously described for β-actin (Garcia-Recio et al., 2013), FSP (Rudnick et al., 2011), CD45 and CD146 (Shipitsin et al., 2007). RNA extraction was performed according to the manufacturer instructions (Qiagen™). cDNA synthesis was carried out using a High-Capacity cDNA Reverse Transcription Kit (Life Technologies™), and qPCR was carried out using SsoAdvanced™ SYBR® Green Supermix (Bio-Rad ™).

## Results and discussion

**Figure 2:**
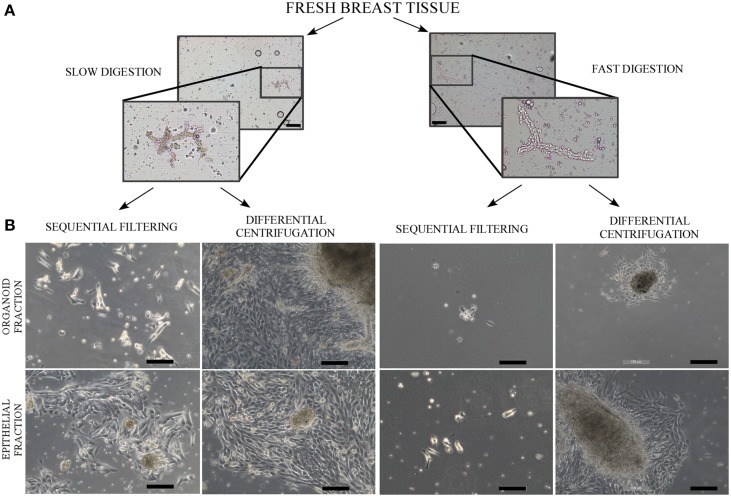
**Phase contrast microscope images of uncultured organoids after digestion and a culture of organoids and epithelial cells**. **(A)** Contrast phase microscopy images from organoids after the slow digestion and fast digestion protocol. The organoids were obtained from RM86 patient. **(B)** Contrast phase microscopy images from organoid and epithelial fractions in culture, 6–8 days after being seeded. The cells correspond to the following patients: RM76 patient for slow digestion and sequential filtering, RM76 patient for slow digestion and differential centrifugation, RM78 patient for fast digestion and sequential filtering and RM81 patient for fast digestion and differential centrifugation. Scale bar: 100 μm.

**Figure 3:**
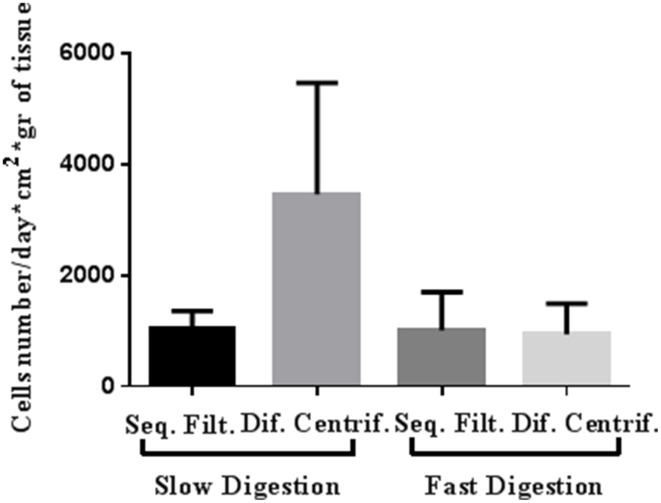
**Representative cell yield of mammary epithelial cells after four approaches to breast tissue digestion and cell fractioning: number of cells grown until the first trypsinization (per day, cm^2^ and g of breast tissue digested)**. The cells were obtained from both organoid and epithelial fractions and from RM104, RM108, and RM109 patients.

Slow digestion with a lower enzymatic concentration helps to obtain a greater number of viable cells that grow and give rise to a greater quantity of cells in culture prior to first trypsinization, starting from both organoid and epithelial cell fractions (Table 1, Figures 2B, 3). Tissue obtained from 15 patients was used for these studies (Table S1). Even though the patients varied in age, parity and pharmacological treatment, this variability did not affect the efficiency of the selected method.

**Table 1:** **Summary of the results obtained using the four technical approaches to breast tissue digestion and cell fractioning, in order to obtain epithelial and myoepithelial cells**.

	**Sequential filtering**	**Differential centrifugation**
Slow digestion	Cell yield: 1040 ± 316 cells/day · cm^2^ ·gr tissue	Cell yield: 3464 ± 2008 cells/day ·cm^2^ ·gr tissue
	Population doubling per day: low	Population doubling per day: high
	Correct 3D structures: yes	Correct 3D structures: yes
	Epithelial cells: low	Epithelial cells: low
	Myoepithelial cells: high	Myoepithelial cells: high The most efficient method
Fast digestion	Cell yield: 1006 ± 692 cells/day ·cm^2^ ·gr tissue	Cell yield: 940 ± 550 cells/day ·cm^2^ ·gr tissue
	Population doubling per day: low	Population doubling per day: medium
	Correct 3D structures: yes	Correct 3D structures: yes
	Epithelial cells: very low	Epithelial cells: very low
	Myoepithelial cells: high	Myoepithelial cells: high

Cell yield is calculated as number of cells grown until the first trypsinization, per day, cm^2^ and g of breast tissue digested (n = 3).

Slow digestion is comparatively less aggressive than fast digestion, since the enzymatic concentration used is smaller and the mechanical handling is less forceful. When tissue is processed using the fast digestion technique, it is chopped and grinded using a food processor, whereas in the slow digestion, it is cut in small pieces using only scissors. Besides, during the fast digestion a magnetic stirrer is used to obtain a homogenized suspension, applying a more forceful agitation. These facts make the method more mechanically aggressive than slow digestion. Thus, cell yield per grams of tissue obtained after fast digestion was always smaller if compared to slow digestion technique (Table 1). Despite the fact that when we measured cell viability after digestion no differences were observed, the smaller cell yield suggest that the digestion step is compromising cell status.

These results are in the same direction of those obtained by Hines et al. (2014) who demonstrated that from slow digestion strategy, 5 times more organoids were obtained compared to fast digestion. In this context, we also observed a great difference between the pellets obtained after fast and slow digestion techniques (Figure S3A). Hines and colleagues concluded that the force and length of agitation were responsible of the digestion efficiency, rather than the enzyme concentration (Hines et al., 2014). Even if our results are in agreement with those researchers, we think that fractionation, the second part of the isolation technique, has also an important responsibility in cell yield.

In fact, slow digestion combined with differential centrifugation (Table 1, Figures 2B, 3) helps to obtain better cell growth and efficiency than when it is combined with the sequential filter method (Table 1, Figures 2B, 3). When the fractionation step is performed by sequential filtering, large organoids are recovered from the upper part of the filters, by cleaning them several times using a pipette. This approach is very aggressive for the organoid fraction, and thus the organoids obtained are smaller and less productive than in differential centrifugation. Therefore, this step could compromise the yield of organoids and their viability compared to differential centrifugation, where organoids are virtually all recovered, as previously described (Speirs et al., 1998). In fact, the pellets obtained after the two fraction techniques showed that differential centrifugation is more efficient than sequential filtering (Figure S3B).

Even if the fast digestion technique generally does not give as good results as slow digestion, when combined with differential centrifugation (Figures 2B, 3) the viability of the cells after seeding is higher than in sequential filtering (Figure 2B). The combination of fast digestion and sequential filtering (Figure 2B) is an approach that makes it more difficult to obtain viable cells when first seeded, regardless of whether the culture was started from the organoid or the epithelial fraction. The combination of slow digestion and differential centrifugation (Table 1, Figures 2B, 3, Figure S4) gave rise to more frequent attachment of larger organoids, together with a greater outgrowth from them. These results were obtained after the analysis of breast tissue collected from 15 patients (Table S1). The variability inherent to each patient is reflected in our results, as it is shown in the standard deviation of the number of cells obtained from each methodology (Table 1, Figure 3). However, the yield obtained in every patient was always in agreement with the results shown in Table 1 and in Figure 3, Figure S4.

**Figure 4:**
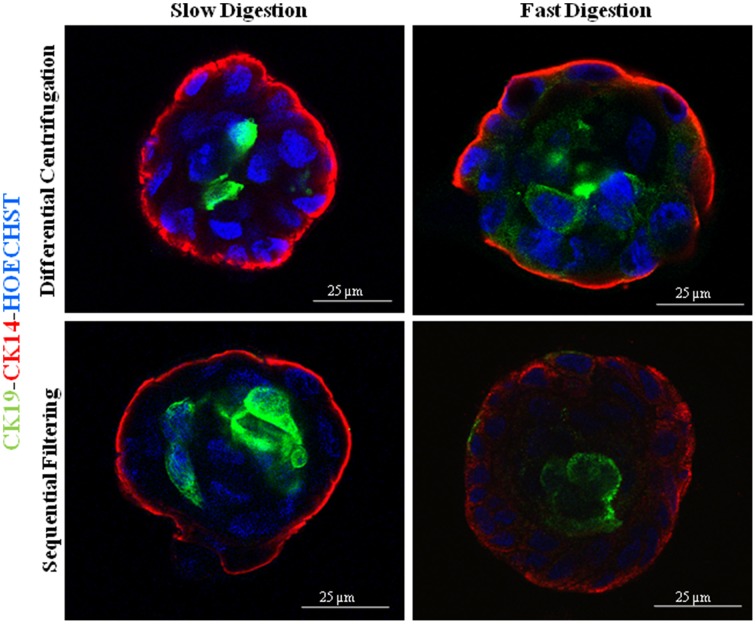
**Immunofluorescence images of 3D cultures by confocal microscopy for the different methodological strategies performed: K14 (myoepithelial cells), K19 (epithelial cells) and nuclei counterstained with Hoechst**. The cells were obtained from the following patients: RM90 patient for slow digestion and differential centrifugation, RM104 patient for fast digestion and differential centrifugation, RM104 patient for slow digestion and sequential filtering and RM19 patient for fast digestion and sequential filtering. Scale bar: 25 μm.

**Figure 5:**
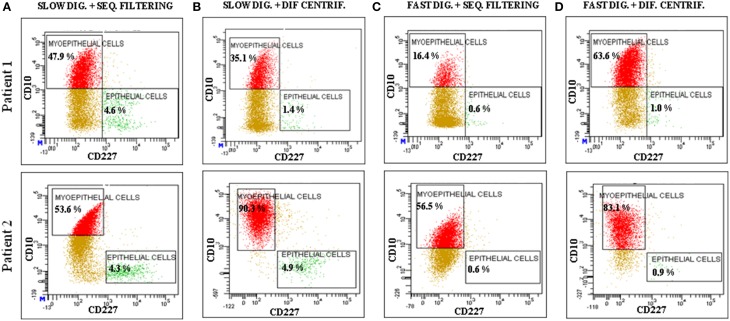
**Analysis of the expression of CD10 and CD227 by flow cytometry**. The analyzed cells were obtained from two patients (Patient 1: RM108 and Patient 2: RM109), through a combination of the following techniques: **(A)** slow digestion and sequential filters, **(B)** slow digestion and differential centrifugation, **(C)** fast digestion and sequential filters and **(D)** fast digestion and differential centrifugation.

Regardless of the technique investigated, once the cells started to grow they were able to form proper acinar structures when seeded on top of matrigel cultures, since myoepithelial cells (K14^+^) were located in the edges surrounding the epithelial cells (K19^+^) (Figure 4). Therefore, all the methods described here can provide viable cells (Speirs et al., 1998; Gudjonsson et al., 2002, 2004; Stingl et al., 2005; Shipitsin et al., 2007; Raouf and Sun, 2013). Moreover, the presence of both populations found in normal breast tissue, myoepithelial (CD10^+^) and epithelial (CD227^+^) cells, was analyzed by flow cytometry (Figure 5). It was found that the myoepithelial cell fraction is larger than the epithelial one. Even though the differences were not significant, a higher number of epithelial cells tended to be obtained when slow digestion was used (Figures 5A,B). This trend was found in every sample analyzed. In this context, it has been described that some markers such as CD44 are lost due to tissue dissociation protocol (Hines et al., 2014). Even if CD227 and CD10 proteins have not been reported to be disappeared, the fact that CD227^+^ cells are lost when fast digestion is performed, suggested that this antigen could be suffering from a similar phenomenon described for CD44 (Hines et al., 2014).

The qPCR (Figure S1) indicated that the primary cells in the culture were not contaminated by other cell types that are present in the mammary gland, such as fibroblasts, endothelial cells or lymphocytes.

**Figure 6:**
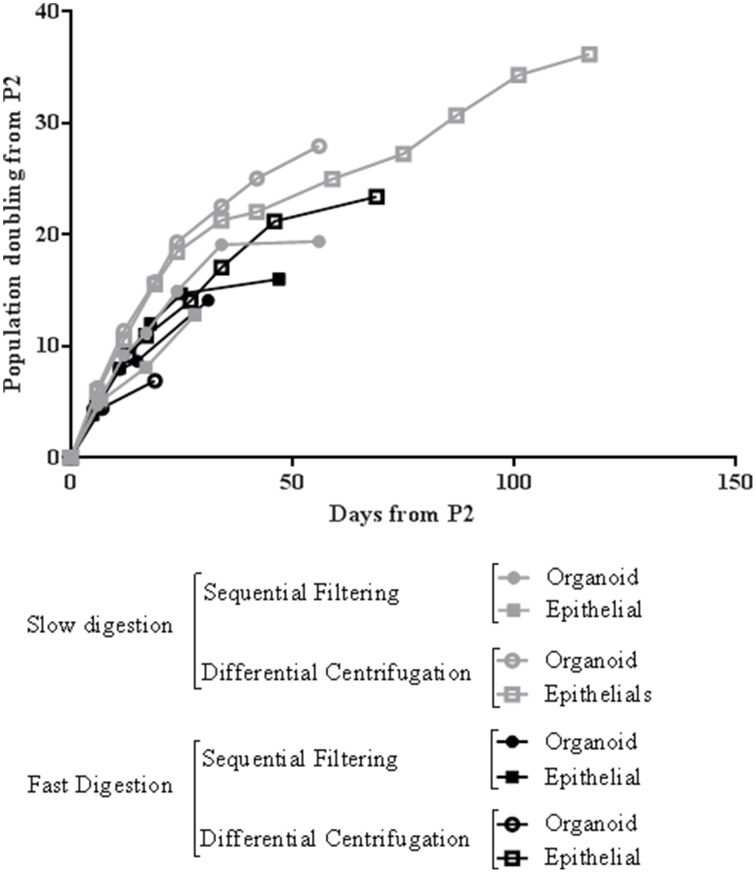
**Representative growth curve of mammary epithelial cells from four methods of breast tissue digestion and cell fractioning after the second passage**. The cells used for this growth curve were obtained from RM109 patient.

Regarding cell growth, the general idea inferred from the imaging analysis was confirmed by the study of growth curves. Slow digestion combined with the differential centrifugation technique gave rise to cells that presented higher population doublings per day than cells from the other methods studied. In every case analyzed, the growth curve was exponential. However, cells obtained by slow digestion coupled with differential centrifugation had a steeper slope (Figure 6), which indicates a faster population doubling time. The mechanical aggressiveness described for both fast digestion (Hines et al., 2014) and sequential filtering could be compromising cell status, affecting to their growth. In fact, fast digestion followed by sequential filtering was the method in which the cells took the most time to grow and double when first seeded after the digestion and fractioning (Figure 2B). However, once trypsinized, their growth speed was similar to that of cells obtained using the other techniques (with the exception of slow digestion followed by differential centrifugation) (Figure 6). In fact, regardless of the method, all the cells were able to grow and maintain their functional characteristics, as proved by other groups (Speirs et al., 1998; Gudjonsson et al., 2002; Stingl et al., 2005; Ince et al., 2007; Shipitsin et al., 2007; Labarge et al., 2013). The present results demonstrate that a combination of slow digestion and differential centrifugation is the best method to obtain efficiently more viable cells that preserve lineage specific antigens and functional features.

## Conclusions

In order to understand breast cancer etiology, it is crucial to comprehend the behavior of the cells in the normal human mammary breast gland. For this purpose, it is important to work with primary epithelial cells, since commercially sold cells present genetic alterations that distance them from the cells found in normal breast tissue (Burdall et al., 2003). However, low yield is a serious limitation to performing research with breast epithelial primary cells. Therefore, it is important to optimize the method, to obtain epithelial and myoepithelial cells from reduction mammoplasties with high viability, whilst preserving the antigens present in normal tissue. In this context, our studies demonstrate that slow digestion of the mammary tissue followed by the differential centrifugation technique is the approach that best fits these requirements. In addition, we found it to be the most cost-effective method. The cells obtained using this method were the most viable in culture, had the most delayed senescence, and maintained the antigens found in both main lineages that are present in breast tissue: epithelial and myoepithelial cells. Furthermore, the long viability of the cells allows us to perform further experiments to separate these two populations if we wish to study them independently. We believe that this optimized method represents a major improvement for the proper isolation of epithelial and myoepithelial cells, and provides the scientific community with the most efficient method in terms of cell yield and viability.

### Conflict of interest statement

The authors declare that the research was conducted in the absence of any commercial or financial relationships that could be construed as a potential conflict of interest.

## Supplementary material

Table S1 **List of samples from 15 patients submitted to technical strategies to perform this methodological implementation.** Information about the technique/s carried out, as well as additional data from each patient.

Figure S1 **qPCR analysis of mRNA expression of endothelial (CD146), lymphocytic (CD45) and fibroblastic (FSP) markers related to the corresponding control sample.** Data are obtained from RM109 patient and 2-ΔΔCt method and presented as mean ± SEM (*n* = 3).

Figure S2 **Flowchart and table describing the fractions recovered from the upper part and the flow-through of each filter used.** Organoids correspond to epithelial and myoepithelial cells and single cells consist of epithelial, myoepithelial and stromal cells.

Figure S3 **Representative image of pellets obtained after digestion and fractioning techniques of two different patients.** **(A)** Representative image after fast digestion and slow digestion (RM109 patient). **(B)** Representative image of pellets from both organoid and epithelial fractions after sequential filtering and differential centrifugation (RM108 patient).

Figure S4 **Cell yield of mammary epithelial cells (organoid and epithelial fractions) after four approaches of breast tissue digestion and cell fractioning: number of cells grown until the first trypsinization (per day, cm^2^ and g of breast tissue digested)**. **(A)** RM108 and **(B)** RM109 patients.
