# Supplementary material for: Comparison of methods for the isolation of human breast epithelial and myoepithelial cells
Source: Front Cell Dev Biol. 2015 May 21;3:32. doi: 10.3389/fcell.2015.00032 (PMC4440402; doi:10.3389/fcell.2015.00032)
Supplement: Table S1 — List of samples from 15 patients submitted to technical strategies to perform this methodological implementation. Information about the technique/s carried out, as well as additional data from each patient. [file Table1.PDF]

Patient	Digestion Technique	Fractionation Strategy	Age	Parity	Health	Medication	Familiar Medical History
**RM1**	Fast	Sequential Filtering	35	2	Ok		
**RM19**	Fast	Sequential Filtering	23	0	Ok		
**RM69**	Slow	Differential Centrifugation	60	0	Ok		
**RM73**	Slow	Differential Centrifugation	27	1	Ok		
**RM76**	Slow	Sequential Filtering	39	2	Ok		
		Differential Centrifugation					
**RM78**	Fast	Sequential Filtering	29	0	Ok		Grandmother and aunt with breast cancer
	Slow						
**RM81**	Slow	Differential Centrifugation	44	1	Ok		
	Fast						
**RM86**	Slow	Sequential Filtering	38	2	Ok		
	Fast						
**RM90**	Slow	Differential Centrifugation	38	2	Ok		
	Fast						
**RM91**	Slow	Differential Centrifugation	41	2	Contralateral breast cancer	EC90 x 4 cycles and taxotere	Sister and aunts with breast cancer
**RM101**	Slow	Sequential Filtering	20	0	Ok		
	Fast	Differential Centrifugation					
**RM102**	Slow	Sequential Filtering	33	1	Contralateral breast cancer	Tamoxifen and taxotere	
		Differential Centrifugation					
**RM104**	Slow	Sequential Filtering	32	0	Ok		
	Fast	Differential Centrifugation					
**RM108**	Slow	Sequential Filtering	23	0	Ok		
		Differential Centrifugation					
	Fast	Sequential Filtering					
		Differential Centrifugation					
**RM109**	Slow	Sequential Filtering	41	0	Ok		
		Differential Centrifugation					
	Fast	Sequential Filtering					
		Differential Centrifugation					
