# Supplementary figures and images for: Comparison of methods for the isolation of human breast epithelial and myoepithelial cells
Source: Front Cell Dev Biol. 2015 May 21;3:32. doi: 10.3389/fcell.2015.00032 (PMC4440402; doi:10.3389/fcell.2015.00032)

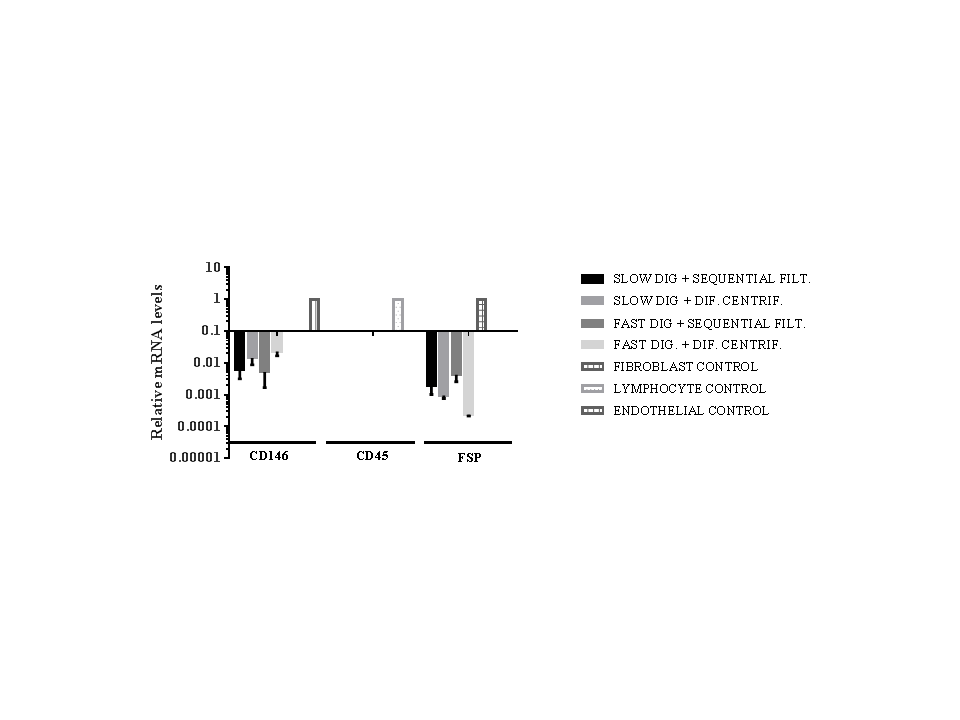

Supplement: Figure S1 — qPCR analysis of mRNA expression of endothelial (CD146), lymphocytic (CD45) and fibroblastic (FSP) markers related to the corresponding control sample. Data are obtained from RM109 patient and 2-ΔΔCt method and presented as mean ± SEM (n = 3). [file Image1.TIFF]

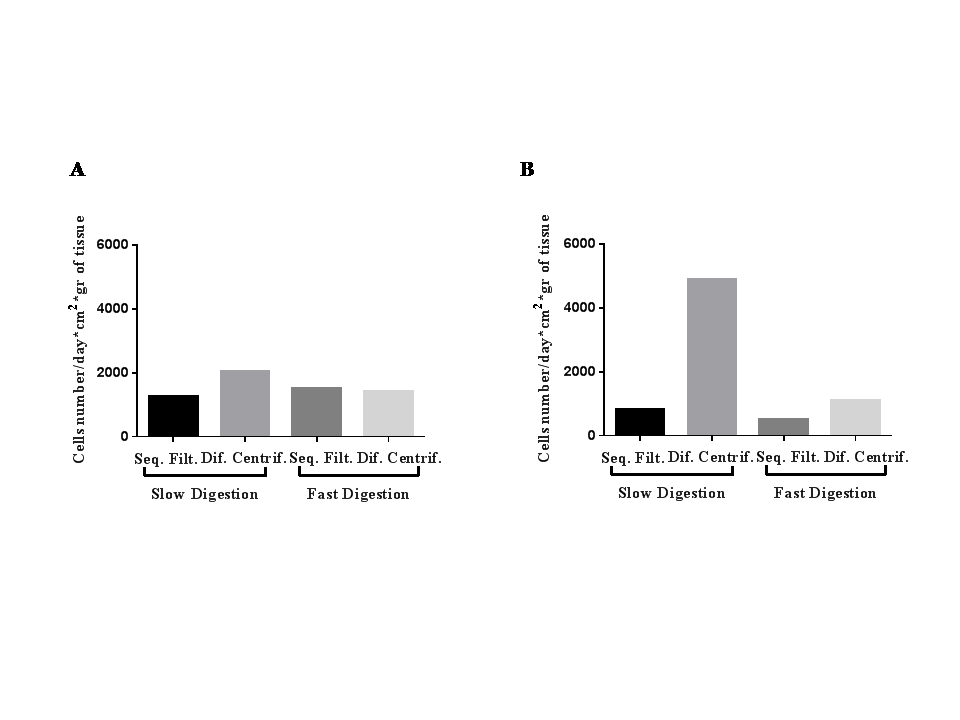

Supplement: Figure S4 — Cell yield of mammary epithelial cells (organoid and epithelial fractions) after four approaches of breast tissue digestion and cell fractioning: number of cells grown until the first trypsinization (per day, cm2 and g of breast tissue digested). (A) RM108 and (B) RM109 patients. [file Image4.TIF]
