# Supplementary material for: Comparison of methods for the isolation of human breast epithelial and myoepithelial cells
Source: Front Cell Dev Biol. 2015 May 21;3:32. doi: 10.3389/fcell.2015.00032 (PMC4440402; doi:10.3389/fcell.2015.00032)
Supplement: Figure S2 — Flowchart and table describing the fractions recovered from the upper part and the flow-through of each filter used. Organoids correspond to epithelial and myoepithelial cells and single cells consist of epithelial, myoepithelial and stromal cells. [file Image2.PDF]

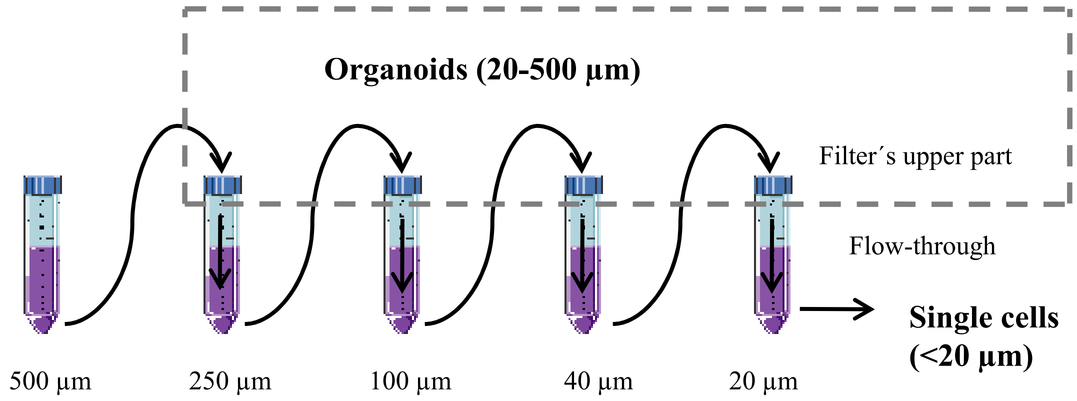

Filters size		Single cells (<20 µm)	Organoids 20-500 µm
250 µm	Upper part		x
	Flow-Through		
100 µm	Upper part		x
	Flow-Through		
40 µm	Upper part		x
	Flow-Through		
20 µm	Upper part		x
	Flow-Through	x	
