# Supplementary material for: Comparison of methods for the isolation of human breast epithelial and myoepithelial cells
Source: Front Cell Dev Biol. 2015 May 21;3:32. doi: 10.3389/fcell.2015.00032 (PMC4440402; doi:10.3389/fcell.2015.00032)
Supplement: Figure S3 — Representative image of pellets obtained after digestion and fractioning techniques of two different patients. (A) Representative image after fast digestion and slow digestion (RM109 patient). (B) Representative image of pellets from both organoid and epithelial fractions after sequential filtering and differential centrifugation (RM108 patient). [file Image3.PDF]

A

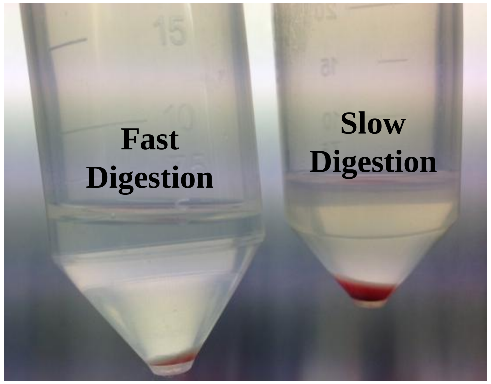
Fast Digestion
Slow Digestion

B

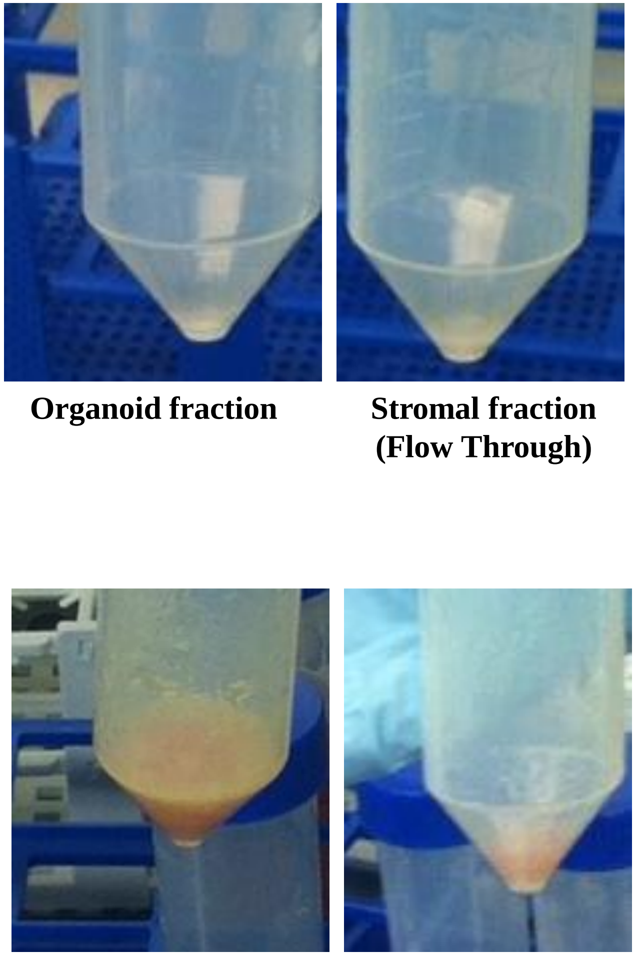
Sequential Filtering
Organoid fraction
Stromal fraction (Flow Through)
Differential Centrifugation
Organoid fraction
Epithelial fraction
